# Herpesviral microRNAs in Cellular Metabolism and Immune Responses

**DOI:** 10.3389/fmicb.2017.01318

**Published:** 2017-07-18

**Authors:** Hyoji Kim, Hisashi Iizasa, Yuichi Kanehiro, Sintayehu Fekadu, Hironori Yoshiyama

**Affiliations:** Department of Microbiology, Faculty of Medicine, Shimane University Shimane, Japan

**Keywords:** microRNA, herpesvirus, oncogenesis, immune evasion, cell metabolism

## Abstract

The microRNAs (miRNAs) function as a key regulator in many biological processes through post-transcriptional suppression of messenger RNAs. Recent advancements have revealed that miRNAs are involved in many biological functions of cells. Not only host cells, but also some viruses encode miRNAs in their genomes. Viral miRNAs regulate cell proliferation, differentiation, apoptosis, and the cell cycle to establish infection and produce viral progeny. Particularly, miRNAs encoded by herpes virus families play integral roles in persistent viral infection either by regulation of metabolic processes or the immune response of host cells. The life-long persistent infection of gamma herpes virus subfamilies, such as Epstein-Barr virus and Kaposi's sarcoma-associated herpesvirus, induces host cells to malignant transformation. The unbalanced metabolic processes and evasion from host immune surveillance by viral miRNAs are induced either by direct targeting of key proteins or indirect regulation of multiple signaling pathways. We provide an overview of the pathogenic roles of viral miRNAs in cellular metabolism and immune responses during herpesvirus infection.

## Introduction

The microRNAs (miRNAs) are small non-coding RNAs consisting of 19–23 nucleotides. The primary miRNA in the nucleus is cleaved into smaller pre-miRNA consisting of around 70 nucleotides with a hairpin structure. The pre-miRNA is then exported to the cytoplasm, where it is cleaved by Dicer to form mature miRNA. The miRNA is incorporated into the RNA-induced silencing complex (RISC), which contains the essential endonuclease Argonaute 2 (Ago2). The miRNA-RISC interacts with the 3′ untranslated region (UTR) in mRNA. This complex suppresses target gene expression through the translational repression or induction of mRNA deadenylation (Winter et al., 2009). Cellular miRNAs play important parts in the regulation of cellular pathways, of which dysregulation has been linked to many disorders including cancer. Viral miRNAs were reported originally by Pfeffer et al. (2004), and now many DNA viruses are known to contain miRNAs in their genomes. More than 200 viral miRNAs are currently identified mainly in the herpesvirus family (Table 1).

**Table 1:** The role of herpes virus-encoded miRNAs in immune evasion and cancer metabolism.

**Viruses**	**miRNAs**	**Targets**	**Target sites (Upper: Target, Lower: miRNA)**	**Function**	**References**
EBV	miR-BART16	LMP1	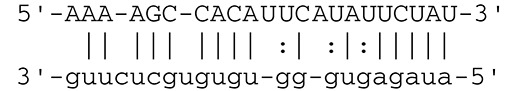	Latent membrane protein that mimics CD40 and induces NF-κB activation	Lo et al., 2007
			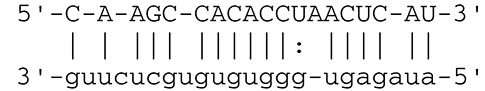		
	miR-BART17-5p	LMP1	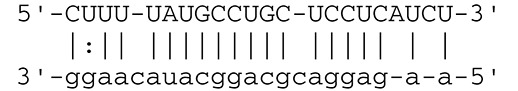	Latent membrane protein that mimics CD40 and induces NF-κB activation	Lo et al., 2007
			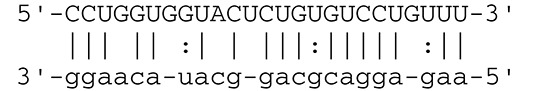		
			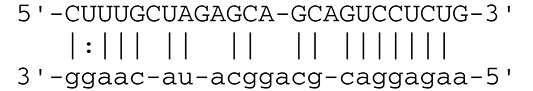		
	miR-BART1-5p	LMP1	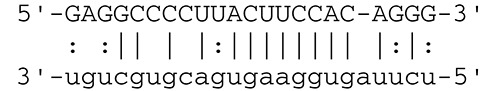	Latent membrane protein that mimics CD40 and induces NF-κB activation	Lo et al., 2007
			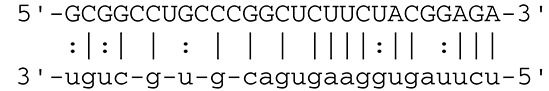		
			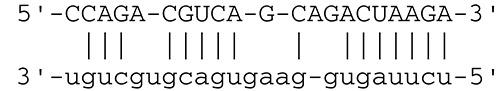		
	miR-BART22	LMP2A	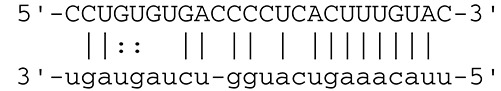	Latent membrane protein that mimics B cell receptor	Lung et al., 2009
	miR-BART2-5p	BALF5	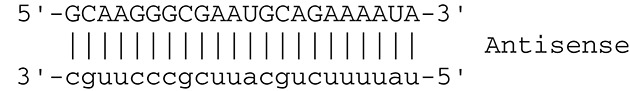	Viral DNA polymerase in lytic infection	Barth et al., 2008
	miR-BART2-5p	MICB	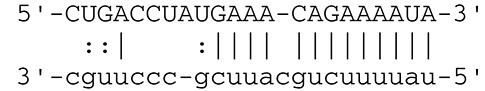	Cellular stress-induced ligand for (NKG2D type II receptor NK cell, CD8 αβT cell)	Nachmani et al., 2009
			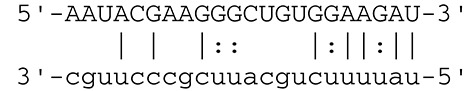		
	miR-BART3	IPO7	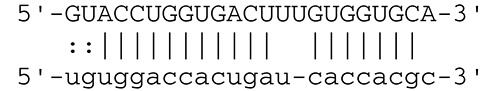	Nuclear import receptor relating to innate immunity	Dolken et al., 2010
	miR-BART15	NLRP3	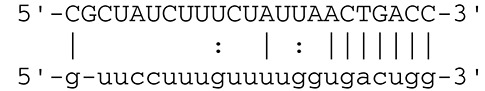	Component of inflammasome	Haneklaus et al., 2012
	miR-BART20-5p	BZLF1	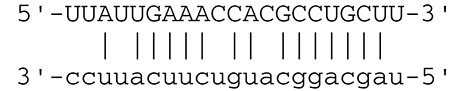	Immediate-early genes that induce lytic replication	Jung et al., 2014
		BRLF1	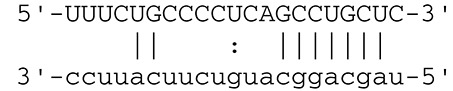		
	miR-BART6-5p	Dicer1	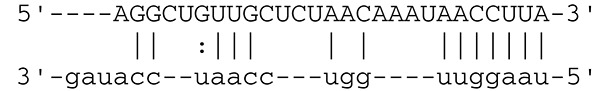	RNase III family enzyme generating miRNA from pre-miRNA	Iizasa et al., 2010
			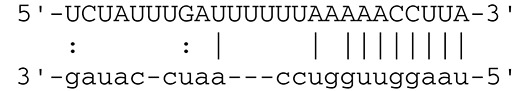		
			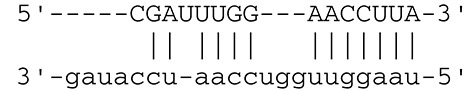		
			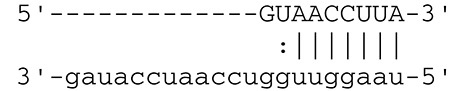		
	miR-BHRF1-3	CXCL11	Not defined in the reference	Chemoattractant factor for activated T cells (chemokine)	Xia et al., 2008
		(I-TAC)			
	miR-BART1	PHGDH	Not defined in the reference	Central metabolite involved in synthesis of L-serine, cystine, and glycine	Ye et al., 2013
		EHD1	Not defined in the reference	Regulator of endosomal transport of plasma membrane proteins	
		PTEN	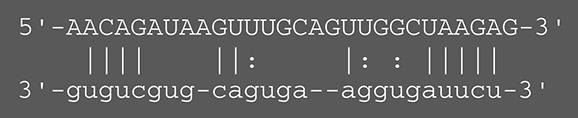	Tumor suppressor gene antagonizing the PI3K/AKT pathway	Cai et al., 2015
			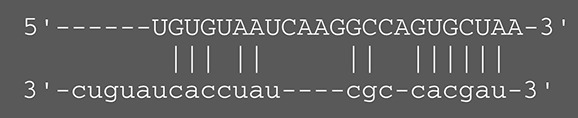	
	miR-BART1-5p,3,4,5,6,7,8,10,18-3p	TGF-β	Not defined in the reference	Metabolic reprogramming	Wan et al., 2015
		Pathway	Not defined in the reference	Regulation of Warburg metabolism	
		Wnt pathway			
	miR-BHRF1	SUMO pathyway	Not defined in the reference	Post-translational modification involved in various cellular processes	Callegari et al., 2014
KSHV	miR-K12-7	MICB	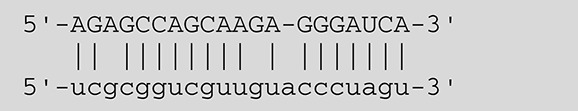	Cellular stress-induced ligand for NKG2D type II receptor (NK cell, CD8 αβT cell)	Nachmani et al., 2009
	KSHV miRNAs	EGLN2	Not defined in the reference	Enzyme involved in the hypoxia-inducible factor signaling pathways	Yogev et al., 2014
		HSPA9	Not defined in the reference	Key protein in mitochondrial import machinery	
HCMV	miR-UL112	MICB	Not defined in the reference	Cellular stress-induced ligand for NKG2D type II receptor (NK cell, CD8 αβT cell, γδT cell)	Nachmani et al., 2010
	miR-US4-1	ERAP1	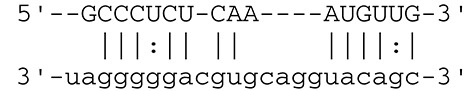	Aminopeptidase 1 processing protein to form HLA class I binding peptide	Kim et al., 2011
			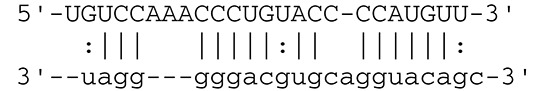		
	miR-UL148D	CCL5 (RANTES)	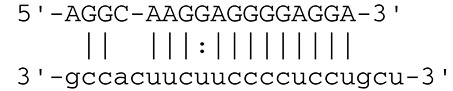	Chemoattractant factor for memory T cells and eosinophils (chemokine)	Kim et al., 2012
	miR-UL112-3p	TLR2	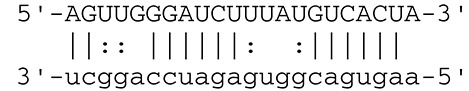	Receptor for peptidoglycan (lipopolysaccharide) activating inflammation	Landais et al., 2015
			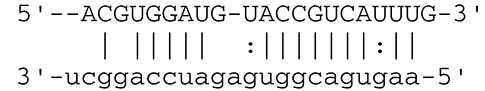		
	miR-UL112-3p	IKKA, IKKB	Not defined in the reference	Inducer of NF-κB signal, upregulation of Glut3 by mediating aerobic glycolysis	Hancock et al., 2017
	miR-US5-1	IKKA, IKKB	Not defined in the reference	Inducer of NF-κB signal, upregulation of Glut3 by mediating aerobic glycolysis	
HSV-1	miR-H8	PIGT	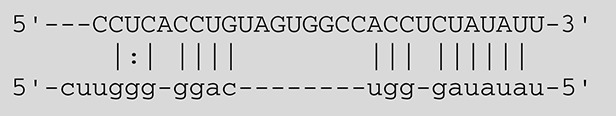	Component of GPI transamidase complex	Enk et al., 2016
	miR-H6	ICP4	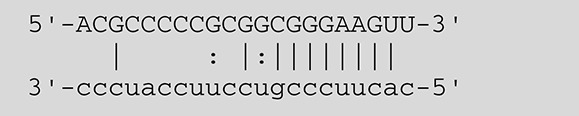	Transactivator of lytic infection-associated genes	Umbach et al., 2008
	miR-H2	ICP0	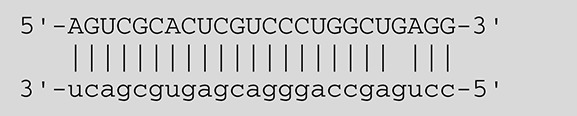	E3 ubiquitin ligase that activates viral gene transcription	Umbach et al., 2008
	miR-H3	ICP34.5	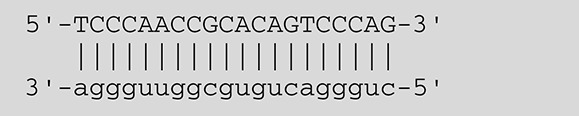	Neurovirulence factor that inactivates autophagy	Umbach et al., 2008
	miR-H4	ICP34.5	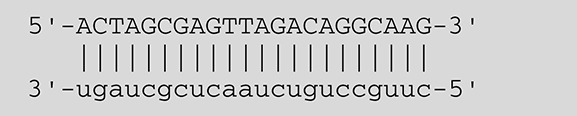	Neurovirulence factor that inactivates autophagy	Umbach et al., 2008
	miR-H4-5p	p16INK4A	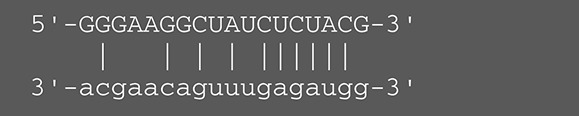	Tumor suppressor gene and repressor of the PI3K/AKT pathway	Zhao et al., 2015

*White or gray backgrounds relate to immune evasion, and black backgrounds relate to cancer metabolism. KSHV, Kaposi's sarcoma-associated herpesvirus; HCMV, human cytomegalovirus; HSV, herpes simplex virus*.

**Figure 1:**
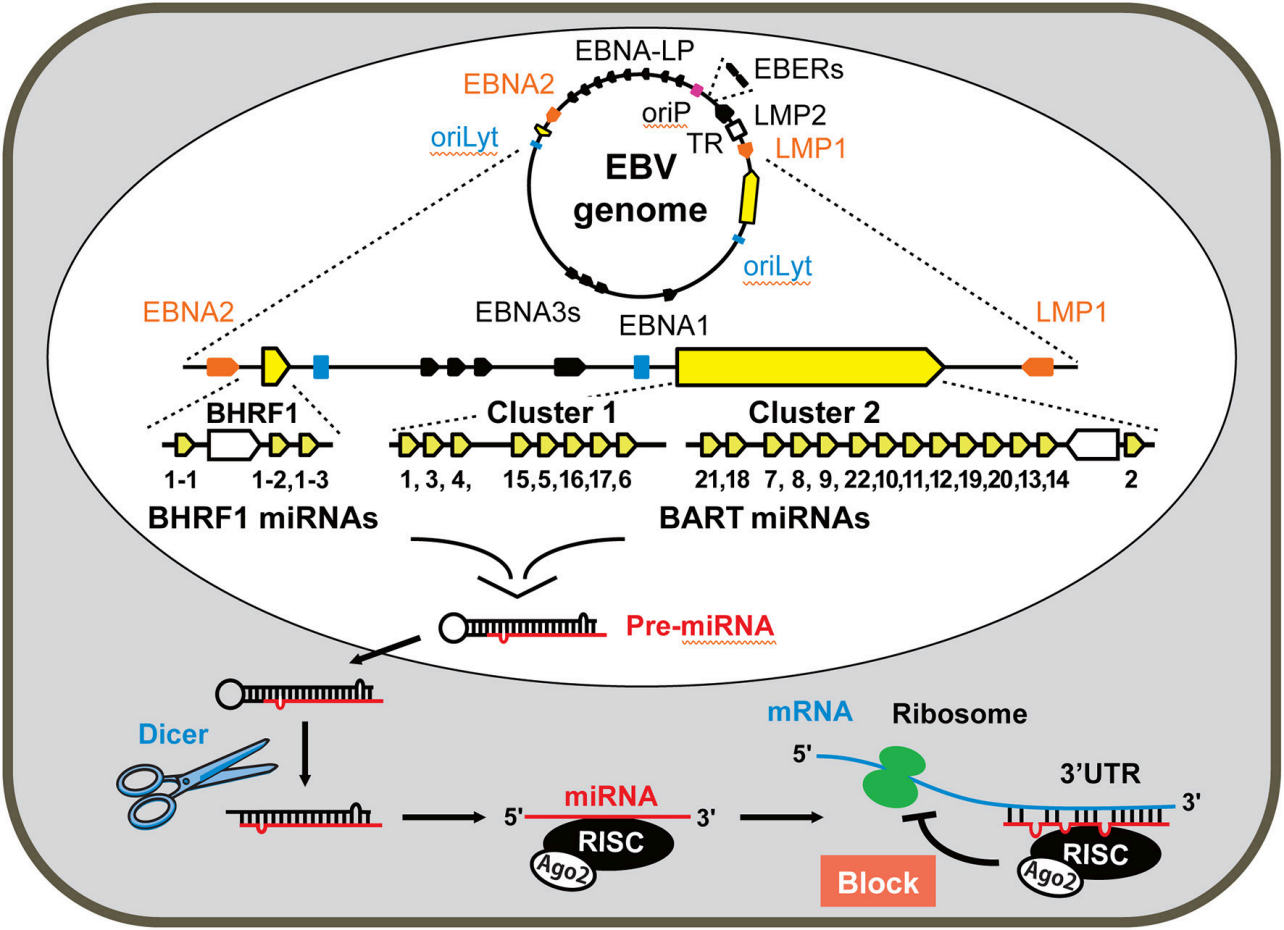
Genome organization and location of miRNAs encoded by the Epstein-Barr virus (EBV). The EBV genome is maintained as a circular episome in latently infected cells. The relative positions of the latency-associated genes on the episomal genome are indicated. The origin of plasmid replication (oriP) and two origins of lytic replication (oriLyt) are indicated as a red and two blue squares, respectively. Orange arrows represents the coding regions of the latent membrane protein 1 (LMP1) and EBNA2 transcribed in antisense and sense orientations, respectively. EBNA-LP is transcribed from variable numbers of repetitive exons (denoted by black lines between EBNA2 and oriP). EBNA3s and EBNA1 shown by black arrows locate between two oriLyt regions. The highly transcribed non-polyadenylated EBER RNAs are represented in the right top of the diagram as black arrows. TR (represented by the white square) denotes the terminal repeats of EBV DNA. LMP2 locating between EBERs and TR is transcribed in sense. The genomic location of miRNAs encoded by EBV is enlarged and represented linearly. The regions of the BHRF cluster and BART clusters 1 and 2 are expanded to show individual miRNAs. Each miRNA is processed from pre-miRNA precursors and transported from nucleus to cytoplasm. Dicer is a part of the RNase III enzyme and cleaves pre-miRNA into short double-stranded RNA fragments called miRNA. The complex of RNA-induced silencing complex (RISC), Argonaute 2 (Ago2), and miRNA specifically inhibits transcription of the target mRNA.

The first report of viral miRNA was described in the herpesvirus family, especially in Epstein-Barr virus (EBV) strain B95-8 having a 12-kb deletion in *Bam*HI-A rightward transcripts (BART) region (Pfeffer et al., 2004). Subsequent studies revealed that EBV encodes 44 different mature BART miRNAs and 4 mature *Bam*HI-H rightward open reading frame 1 (BHRF1) miRNAs (Cai et al., 2006; Grundhoff et al., 2006; Zhu et al., 2009) (Figure 1). Rhesus lymphocryptovirus encodes miRNAs, which are orthologs of EBV miRNAs and evolutionarily conserved (Cai et al., 2006). Kaposi's sarcoma-associated herpesvirus (KSHV), the etiologic agent of Kaposi's sarcoma, encodes 13 precursor (pre)-miRNAs. These KSHV miRNAs are located in the KSHV latency-associated region (Pfeffer et al., 2005; Grundhoff et al., 2006). By sharing target genes, these KSHV miRNAs function as analogs of cellular oncomirs (Skalsky et al., 2007; Gottwein et al., 2011; Manzano et al., 2013).

This mini-review summarizes the role of viral miRNAs in cellular metabolism and the immune system of hosts to understand the pathologies of viral infection. Although viral miRNAs have also been detected in JC virus and human immunodeficiency virus, we mainly discuss miRNAs from *Herpesviridae*.

## Pathological roles of EBV miRNAs

### EBV-encoded miRNAs

EBV is associated with many tumors, including lymphoma and epithelial carcinomas (Young and Murray, 2003; Luo et al., 2005). Detection of EBV-encoded small non-coding RNAs (EBERs) by *in situ* hybridization is used as a diagnostic hallmark of EBV infection in tumor cells. EBERs can be detected in a variety of tumors, such as nasal NK/T-cell lymphoma, post-transplant lymphoma, Burkitt's lymphoma, Hodgkin's disease, diffuse large B-cell lymphoma, nasopharyngeal carcinoma (NPC), and gastric carcinoma (Delecluse et al., 2007). Following primary infection, EBV establishes latent infection. Three latency types (I, II, III) are defined depending on the pattern of expression in viral genes (Middeldorp et al., 2003). BART miRNAs are expressed in all latency types, whereas BHRF1 miRNAs are expressed only in type III latency. BHRF1 transcripts encode four mature BHRF1 miRNAs. BART transcripts have two clusters, cluster 1 and cluster 2, which generate 44 mature BART miRNAs (Figure 1) (Pfeffer et al., 2004; Cai et al., 2006; Zhu et al., 2009). EBV B95-8 strain has a 12-kb deletion and lacks most of the BART miRNAs (Baer et al., 1984).

BART miRNAs are expressed more strongly in EBV-associated epithelial cells than in B lymphocytes (Chen et al., 2010). BART transcripts have two TATA-less promoter regions, designated P1 and P2 (Sadler and Raab-Traub, 1995). Although P1 supports substantial activity in epithelial cells and B lymphocytes, P2 exhibits strong activity only in epithelial cells. Several transcription factors, important for cellular metabolism and immunity, regulate BART promoter activity (Chen et al., 2005). P1 activity is negatively regulated by interferon regulatory factor 5 (IRF5) and IRF7, and P2 activity is positively regulated by c-Myc and CCAAT-enhancer-binding protein (C/EBP) family members. Relative expression between positive and negative transcription factors in EBV-infected cells probably controls the expression level of BART miRNAs. BHRF1 miRNAs are generated as part of the Cp- and/or Wp-initiated EBNA transcripts in cells showing latency III infection (Amoroso et al., 2011). The expression of miR-BHRF1-1 absolutely depends on Cp/Wp activity, because it locates at the 5′ UTR of BHRF1 mRNA and overlaps with the EBV replication-activated BHRF1 promoter (Amoroso et al., 2011). However, because miR-BHRF1-2 and miR-BHRF1-3 locate at the 3′ UTR, they are strongly expressed by an alternative promoter for lytic BHRF1 transcript during lytic replication (Kelly et al., 2009; Xing and Kieff, 2011). The expression of BART miRNAs inversely correlates with the methylation status of the promoter in EBV-infected B lymphocytes. Promoter methylation may be important in regulating the expression of BART and BHRF1 miRNAs (Kim do et al., 2011).

EBV miRNAs are transferred to adjacent cells via exosomes (Pegtel et al., 2010). The secreted viral miRNAs modify the expression of target genes in recipient cells (Haneklaus et al., 2012). Some BART miRNAs show distinctive expression between cells and exosomes. MiR-BART7 is expressed more abundantly in exosomes than in EBV-positive NPC cells (Meckes et al., 2010). However, miR-BART8-5p is expressed less abundantly in exosomes than in lymphoblastoid cell lines (Hoshina et al., 2016). These findings indicate that some EBV miRNAs are selectively packaged and transported into recipient cells via exosomes.

### Immune evasion by EBV miRNAs

EBV can establish either latent infection or lytic replication (Kenney and Mertz, 2014). In tumor cells, EBV usually maintains latent infection rather than entering into lytic replication (Cohen, 2000). During latent infection, EBV transcribes viral miRNAs to escape from the host immune system by targeting both cellular and viral genes. Lowered expression of viral proteins enables infected cells to escape antigenic recognition by the host immune system. Expression of latent membrane protein 1 (LMP1) and LMP2A is downregulated by BART cluster 1 miRNAs (miR-BART16, 17-5p, and 1-5p) (Lo et al., 2007) and miR-BART22 (Lung et al., 2009), respectively. LMP1 and LMP2A are oncogenic viral proteins that promote EBV-positive malignancies by engaging a number of signal pathways, such as the NFκB, JNK/p38-SAPK, PI3K/Akt, ERK-MAPK, and JAK/STAT pathways, followed by subsequent induction of morphological and phenotypic alterations (Young and Rickinson, 2004). LMP1 and LMP2A alter the host immune system by cooperating with environmental and host genetic factors (Dawson et al., 2012).

Viral miRNAs help maintain latent infection by expressing limited numbers of viral genes that allow EBV to evade host immune surveillance. MiR-BART20-5p suppresses lytic replication by targeting EBV immediate-early genes BZLF1 and BRLF1 (Jung et al., 2014), key regulators of the expression of EBV proteins and the production of progeny virus (Pattle and Farrell, 2006). Downregulation of Dicer by miR-BART6-5p reduces the expression of BZLF1, BRLF1, and both EBNA2 and LMP1 latent proteins in C666-1, an EBV-positive NPC cell line (Iizasa et al., 2010). Likewise, miR-BART2-5p blocks lytic replication by inhibiting expression of viral DNA polymerase BALF5 (Barth et al., 2008).

EBV miRNAs can also block immune response by reducing cytokines, chemokines, and T-cell stimulatory molecules. MiR-BHRF1-3 modulates host interferon (IFN) response by targeting CXCL11, an IFN-inducible T-cell-attracting chemokine (Xia et al., 2008). Suppression of MHC class I-related chain B (MICB) by miR-BART2-5p protects EBV-infected cells from attack by NK cells and T cells. MICB is also a target gene for other herpesvirus miRNAs, including KSHV-encoded miR-K12-1 and human cytomegalovirus (HCMV)-encoded miR-UL112 (Nachmani et al., 2009). Importin 7 (IPO7), a receptor for importing transcription factors into the nucleus, has important function for innate immunity, because loss of IPO7 in macrophages inhibits interleukin (IL)-6 secretion (Yang et al., 2009). IPO7 is a putative target for miR-BART1-3p and miR-BART3 (Dolken et al., 2010). MiR-BART15 also reduces IL-1β production from the inflammasome by targeting NLRP3 (NLR family, pyrin domain containing 3; also known as cryopyrin) at the same 3′ UTR region that host miR-223 recognizes (Haneklaus et al., 2012). These findings suggest that EBV miRNAs inhibit immune response by multiple mechanisms.

### EBV miRNAs in cancer metabolism

Compared with normal cells, cancer cells increase metabolic autonomy, which uses nutrients and promotes metabolic processes of the host cells to support proliferation. Recent studies showed that several EBV miRNAs regulate cellular metabolic processes. MiR-BART1 is one of the metabolic regulators strongly expressed in NPC. The expression levels of phosphoglycerate dehydrogenase (PHGDH) and EH domain-containing protein 1 (EHD1) were significantly upregulated by miR-BART1 expression in an NPC cell line, CNE1 (Ye et al., 2013). PHGDH is important for the synthesis of serine and glycine, a central metabolite for a variety of biosynthetic pathways, by removing 3-phosphoglycerate during glycolysis (DeBerardinis, 2011). EHD1 regulates endosomal transport of plasma membrane proteins such as transferrin receptor and ß1 integrin (Jovic et al., 2007).

Wan et al. reported that 9 EBV miRNAs (miR-BART1-5p, 3, 4, 5, 6, 7, 8, 10, and 18-3p) are highly expressed in NPC tissues. Genome pathway analysis indicated that upregulated EBV miRNAs mainly target transforming growth factor β (TGF-β) and Wnt signaling pathways (Wan et al., 2015), which are involved in many biological processes during oncogenesis, including reprogramming of tumor cell bioenergetics (Sherwood, 2015) and the bioenergetic shift toward catabolism (Guido et al., 2012). Previous studies showed that both pathways are dysregulated in NPC (Zeng et al., 2007; Chen et al., 2009). Consistent with this result, the TGF-β signaling pathway is suppressed by upregulation of miR-BHRF1, because miR-BHRF1 targets the small ubiquitin-like modifier-regulated component SMAD3 and the transcription co-regulators JUN and FOS (Callegari et al., 2014). Recently, target genes for EBV miRNAs were identified by high-throughput sequencing of RNA isolated by the methods of crosslinking immunoprecipitation (HITS-CLIP) and photoactivatable ribonucleoside-enhanced crosslinking and immunoprecipitation (PAR-CLIP). These EBV miRNA targets include many components of the Wnt signaling pathway (Riley et al., 2012; Skalsky et al., 2012).

The PI3K/AKT/mTOR pathway is a critical regulator in cell survival, growth, protein synthesis, and glucose metabolism (Yap et al., 2008). Moreover, the AKT pathway promotes the expression of genes involved in glycolysis and lipid genesis (Wullschleger et al., 2006). In NPC cells, miR-BART1 significantly reduces phosphatase and tensin homolog (PTEN) expression while increasing the phosphorylation level of pAKT, pFAK, p130Cas, pShc, and pERK1/2 (Cai et al., 2015). PTEN inhibits the PI3K/AKT pathway by dephosphorylating PIP3 and increasing PIP2, resulting in a reduction of membrane recruitment of AKTs (Rafalski and Brunet, 2011). Thus, miR-BART1 activates migration, invasion, and metastasis of NPC cells via suppression of PTEN (Cai et al., 2015).

MiR-BART7 is expressed strongly in NPC cells and promotes cell proliferation, migration, and invasion. Pathway analysis indicates that the expression level of various genes is altered by the expression of miR-BART7. These target genes belong to the signaling pathway of calcium and the immune system, ionotropic glutamate receptor, ATP-binding cassette transporters, nuclear receptors in lipid metabolism and toxicity, the TGF-ß-signaling pathway, and metabolism of lipids and lipoproteins (Chan et al., 2012). Therefore, the aberrant expression of EBV miRNAs may contribute to metabolic abnormality and oncogenesis in EBV-infected cells by unbalancing various signaling pathways.

## miRNAs in other herpesviruses

### Herpesvirus-encoded miRNAs

Herpesviruses other than EBV and KSHV also encode miRNAs. Herpes simplex virus (HSV) has two serotypes, HSV-1 and HSV-2, which infect oral or genital mucosa. The latency-associated transcript functioning as a primary miRNA precursor is exclusively expressed during latent infection (Wagner et al., 1988; Umbach et al., 2008). Since the first report in 2006, 27 mature miRNA sequences have been identified in the HSV-1 genome (Cui et al., 2006; Jurak et al., 2010). Similarly, HSV-2 encodes 24 mature miRNAs (Umbach et al., 2010). Several miRNAs are conserved between HSV-1 and HSV-2, especially in their seed regions. These viral miRNAs have analogous functions in immune evasion and virus propagation (Jurak et al., 2010; Umbach et al., 2010).

Unlike other herpesviruses, HCMV miRNAs are not clustered in latent transcripts, but are distributed throughout the viral genome (Buck et al., 2007). Currently, 26 mature HCMV miRNA sequences are uploaded on miRBase (http://www.mirbase.org). HCMV miRNAs target multiple genes related to immune response, cell cycle control, and vesicle trafficking (Hook et al., 2014).

Varicella-zoster virus (VZV) is a pathogenic human virus that causes chicken pox and shingles. Unlike other herpesviruses examined, although many small RNA sequencing studies have been performed, VZV miRNAs have not been identified yet (Umbach et al., 2009).

Human herpesvirus 6 (HHV-6), with its two variants HHV-6A and HHV-6B, is a ubiquitous pathogen in general human populations. Both are very closely related, with nearly 90% homology at the genomic level. Deep sequencing of small RNA species identified a small non-coding RNA with the characteristics of a viral miRNA from cells harboring HHV-6A. Growth analyses of mutant viruses revealed that miR-U86 directly impacts lytic replication by targeting HHV-6A immediate-early gene U86 (Nukui et al., 2015). HHV-6B encodes four pre-miRNAs expressed from direct repeat regions located at either side of the genome. HHV-6B miR-Ro6-2 is a seed ortholog of host miR-582-5p, which targets SMAD3 to downregulate TGF-β. HHV-6B miRNAs also have the potential to regulate viral replication (Tuddenham et al., 2012).

HHV-7 is a ubiquitous T-lymphotropic virus infecting most humans. As with VZV, HHV-7-encoded miRNAs have not yet been identified (Louten et al., 2015).

### Herpes viral miRNAs in immune evasion

EBV miR-BART2-5p and KSHV miR-K12-7 target the 3′ UTR of MICB at different locations (Nachmani et al., 2009). HCMV miR-UL112 and cellular miR-376a synergistically downregulate MICB expression and subsequently help the virus evade innate immune recognition (Nachmani et al., 2010). Recognition of HCMV-infected cells by cytotoxic T lymphocytes is impaired due to the reduced expression of aminopeptidase ERAP1 by HCMV miR-US4-1 (Kim et al., 2011). In human fibroblast cells, HCMV clinical strain-specific miR-UL148D was shown to block the human chemokine RANTES, which attracts immune cells during inflammation and the immune response (Kim et al., 2012).

HSV-1 miR-H8 targets the glycosylphosphatidylinositol gene, which results in reduced expression of several immune-modulating proteins, viral expansion, and viral evasion from natural killer cell elimination (Enk et al., 2016). HSV miRNAs also target viral genes to maintain latency and suppress immune function. HSV-1 miR-H6 and miR-H2 reduce infected cell polypeptide 4 (ICP4) and ICP0, respectively (Umbach et al., 2008; Duan et al., 2012). Both miR-H3 and miR-H4 target ICP34.5 mRNA (Umbach et al., 2008). HSV-2 miRNAs also contribute to latency and immune evasion similarly to HSV-1 miRNAs because of the close homology of these two viruses.

In KSHV-infected primary effusion lymphoma cells, KSHV miR-K1 inhibits viral lytic replication by targeting the 3′ UTR of IκBα protein, an inhibitor of the NFκB complexes. Enhanced NFκB activity evades host immune system and promotes cell survival (Lei et al., 2010). The KSHV miRNA cluster also represses a network of targets associated with STAT3 and suppresses STAT3 activation upon IL-6 treatment. KSHV miR-K6-5 targets the 3′ UTR of PKCδ, a Ser/Thr kinase that phosphorylates and activates STAT3. KSHV miR-K921 also targets a second Ser/Tr kinase, IRAK1. Repression of BIRC5, a transcriptional target of STAT3, by KSHV miR-K12-5 promotes KSHV infection. These multiple KSHV miRNAs that repress STAT3 can weaken the innate immune responses to type-I interferons and inhibit the induction of antiviral genes, such as IRF1, IFITM1, and ISG15 (Ramalingam and Ziegelbauer, 2017).

### Herpes viral miRNAs in cancer metabolism

Several core cellular metabolic pathways are significantly altered by herpesvirus infection and the expression of viral miRNAs (Sanchez and Lagunoff, 2015). HSV-1 miR-H4-5p directly targets cyclin-dependent kinase inhibitor 2A (p16) mRNA in neuroblastoma cell lines. Suppression of miR-H4-5p inhibits cell proliferation, invasion, and progression of the cell cycle via the p16-mediated PI3K-AKT signaling pathway (Zhao et al., 2015). Likewise, HCMV miR-UL112-3p modulates the TLR/IRAK1/NFκB signaling pathway by targeting Toll-like receptor 2 mRNA (Landais et al., 2015). NFκB signaling is known to upregulate the expression of Glut3 in p53-deficient cells (Kawauchi et al., 2008), suggesting that inhibition of NFκB signaling by miR-UL112-3p and miR-US5-1 might suppress aerobic glycolysis (Hancock et al., 2017).

KSHV has been shown to alter host cell energy metabolism by concurrent regulation of two independent pathways. First, KSHV miRNAs stabilize and activate transcription factor HIF1α, a master regulator of cell metabolism, by targeting hypoxia-inducible factor prolyl hydroxylase, EGLN2. Second, downregulation of the mitochondrial heat shock protein A9 (HSPA9) by KSHV miRNAs reduces mitochondrial copy numbers and enhances anaerobic glycolysis (Warburg effect). Downregulation of EGLN2 and HSPA9 allows cell proliferation in a low oxygen condition (Yogev et al., 2014). KSHV miR-K12-11 and miR-K12-3 prevent lytic reactivation by reducing the expression of cellular transcription factors MYB, C/EBPα, and Ets-1, which are reported as activators of the RTA promoter (Plaisance-Bonstaff et al., 2014).

Murine gammaherpesvirus 68 (MHV-68), a natural pathogen of wild rodents, encodes for 14 pre-miRNAs. All MHV-68 miRNAs are located downstream of viral tRNA-like elements and transcribed by RNA polymerase III. Recent research showed that an MHV-68 mutant lacking the expression of all miRNAs results in a higher viral genomic load in the spleen. This report shows that MHV-68 miRNAs contribute to the maintenance of latency *in vivo* (Steer et al., 2016).

The generation and analysis of mutant viruses revealed that MHV-68 miRNAs are dispensable for short-term virus replication but are important for the establishment of lifelong infection in memory B cells. Furthermore, a lack of miRNA expression results in the complete attenuation of lethal disease in a virus-induced pneumonia model, demonstrating a key role for the viral miRNAs in pathogenesis (Feldman et al., 2014).

Similar to EBERs in EBV, MHV-68 encodes non-coding RNAs called TMERs (tRNA-miRNA-encoded RNAs), which are highly expressed in latently infected cells. TMERs harbor a predicted tRNA-like element and two downstream pre-miRNA hairpins and are processed by tRNase Z instead of Drosha, similar to cellular non-coding tRNAs, to generate mature miRNAs. Analysis of individual TMER mutant viruses has shown TMER4 to be a key mediator of virus dissemination. Interestingly, TMER4 miRNA seed sequence mutants do not compromise TMER4 function. These results demonstrate a crucial miRNA-independent function of TMER4 in hematogenous dissemination and the establishment of peripheral latency (Feldman et al., 2016).

## Summary and conclusions

Viral miRNAs play important roles in cancer development and progression by modulating immune response and metabolic circuits. Although further study is necessary to understand the pathogenic significance of viral miRNAs, viral miRNAs can be applied for the diagnosis of cancer, identification of drug targets, and therapeutic use.

## Author contributions

HK wrote the manuscript. SF and YK assisted in creating the table. HI and HY edited the paper and contributed financial assistance.

### Conflict of interest statement

The authors declare that the research was conducted in the absence of any commercial or financial relationships that could be construed as a potential conflict of interest.
